# HERC1 Ubiquitin Ligase Is Required for Hippocampal Learning and Memory

**DOI:** 10.3389/fnana.2020.592797

**Published:** 2020-11-19

**Authors:** Eva M. Pérez-Villegas, Mikel Pérez-Rodríguez, José V. Negrete-Díaz, Rocío Ruiz, Jose Luis Rosa, Guillermo Alvarez de Toledo, Antonio Rodríguez-Moreno, José A. Armengol

**Affiliations:** ^1^Department of Physiology, Anatomy and Cell Biology, Universidad Pablo de Olavide, Seville, Spain; ^2^División de Ciencias de la Salud e Ingenierías, Universidad de Guanajuato, Guanajuato, Mexico; ^3^Department of Biochemistry and Molecular Biology, School of Pharmacy, University of Seville, Seville, Spain; ^4^Instituto de Biomedicina de Sevilla-Hospital Universitario Virgen del Rocío/CSIC/Universidad de Sevilla, Seville, Spain; ^5^Departament de Ciències Fisiològiques, IBIDELL, Universitat de Barcelona, Barcelona, Spain; ^6^Department of Medical Physiology and Biophysics, School of Medicine, University of Seville, Seville, Spain

**Keywords:** autophagy, dendritic spines, glutamatergic input, hippocampus, LTP, synapses

## Abstract

Mutations in the human HERC1 E3 ubiquitin ligase protein develop intellectual disability. The *tambaleante* (*tbl*) mouse carries a *HERC1* mutation characterized by cerebellar ataxia due of adult cerebellar Purkinje cells death by extensive autophagy. Our previous studies demonstrated that both the neuromuscular junction and the peripheral nerve myelin sheaths are also affected in this mutant. Moreover, there are signs of dysregulated autophagy in the central nervous system in the *tbl* mouse, affecting spinal cord motor neurons, and pyramidal neurons of the neocortex and the hippocampal CA3 region. The *tbl* mutation affects associative learning, with absence of short- and long-term potentiation in the lateral amygdala, altered spinogenesis in their neurons, and a dramatic decrease in their glutamatergic input. To assess whether other brain areas engaged in learning processes might be affected by the *tbl* mutation, we have studied the *tbl* hippocampus using behavioral tests, *ex vivo* electrophysiological recordings, immunohistochemistry, the Golgi-Cox method and transmission electron microscopy. The *tbl* mice performed poorly in the novel-object recognition, T-maze and Morris water maze tests. In addition, there was a decrease in glutamatergic input while the GABAergic one remains unaltered in the hippocampal CA1 region of *tbl* mice, accompanied by changes in the dendritic spines, and signs of cellular damage. Moreover, the proportions of immature and mature neurons in the dentate gyrus of the *tbl* hippocampus differ relative to the control mice. Together, these observations demonstrate the important role of HERC1 in regulating synaptic activity during learning.

## Introduction

At the end of the 1980’s, the *tambaleante* (*tbl*) mutant mouse was described as a model of adult cerebellar ataxia, a phenotype caused by the nearly complete loss of cerebellar Purkinje cells (Wassef et al., 1987; Rossi et al., 1995). This Purkinje cell death was later proposed to be a model of autophagy (Dusart et al., 2006), and molecular studies identified the spontaneous Gly483Glu substitution in the RCC1 (Regulator of Chromosome Condensation 1) domain of the HERC1 ubiquitin ligase as the mutation that induces the overexpression of the protein responsible for this cell death (Mashimo et al., 2009).

HERC1 is an ubiquitin ligase of the HECT (Homologous to the E6-AP Carboxyl Terminus) family that belongs to the ubiquitin–proteasome system (UPS) (Sánchez-Tena et al., 2016; Schneider et al., 2018; García-Cano et al., 2019). Alterations to the UPS have been related to a variety of neurodegenerative disorders, such as Alzheimer’s, Huntington’s and Parkinson’s disease (de Vrij et al., 2004; Upadhya and Hegde, 2005; Rubinsztein, 2006; Hegde and Upadhya, 2007; van Tijn et al., 2012; Labbadia and Morimoto, 2015), as well as different types of spinal and muscular atrophy (Ramser et al., 2008; Rusmini et al., 2010, 2015; Deng et al., 2011; Dlamini et al., 2013; Rusmini et al., 2015). Furthermore, mutations in HECT E3 ligases have been linked to the pathogenesis of neuromuscular disorders, Parkinson’s disease and diseases of the autism spectrum, such as Angelman syndrome (for a review see Sánchez-Tena et al., 2016; Sluimer and Distel, 2018). In humans, mutations of *HERC1* cause a polymorphic syndrome with (Nguyen et al., 2015) or without cerebellar affectation (Ortega-Recalde et al., 2015; Aggarwal et al., 2016; Hashimoto et al., 2016; Utine et al., 2017), yet always apparently associated with intellectual disability (Aggarwal et al., 2016) and in some cases related to the autism spectrum (Hashimoto et al., 2016; Utine et al., 2017). Furthermore, proteins with mutations in its RCC1-like domain (RLD) are involved in several other neuropathologies, such as juvenile amyotrophic lateral sclerosis 2 and X-linked *retinitis pigmentosa* (Mashimo et al., 2009).

HERC1 contains two RLD domains and the mutation carried by the *tbl* mice is in the N-terminal RLD domain (Mashimo et al., 2009). The N-terminal RLD domain may act as guanine nucleotide-release factor for ARF proteins (Sánchez-Tena et al., 2016) and by interacting with ARF/Rab GTPases, it influences intracellular vesicle trafficking (Sánchez-Tena et al., 2016). Moreover, HERC1 constitutes a ternary complex with clathrin and the heat shock protein, HSP70 (Rosa and Barbacid, 1997). Since clathrin mediated endocytosis is relevant for synaptic vesicle recycling (Rizzoli and Betz, 2005), alterations to the normal clathrin cycle could interfere with normal synaptic function.

The cerebellum is widely recognized as a key center for motor learning (for a review see Manto and Jissendi, 2012) and evidence is accruing that the cerebellum could play a pivotal role in non-motor learning (Lackey and Sillitoe, 2017). Damage to other brain areas as well as the cerebellum has been reported in several cerebellar mutant mice (see Porras-García et al., 2013) and these mutant mice display cerebellum related spatial learning alterations (for a review see Lalonde, 2002). In fact, we previously reported that the associative memory is also impaired in the adult *tbl* mice, impairment that is correlated to alterations of the dendritic spines on neurons in the lateral amygdala, and to the absence of short-term (STP) and long-term (LTP) potentiation in this nucleus (Pérez-Villegas et al., 2018). Thus, it is reasonable to hypothesize that *tbl* mutation could also affect the hippocampus both morphological and physiologically. Therefore, we have analyzed the *tbl* hippocampus using behavioral tests, histological methods, and electrophysiological *ex vivo* recordings to determine the extent and the physiological relevance of HERC1 ubiquitin ligase.

## Materials and Methods

### Animals

*Tambaleante* mice were obtained by breeding pairs of the *tbl* carrier mice, genotyping the offspring by PCR (Mashimo et al., 2009). Three to 4-month-old male *tbl* mice with a fully developed cerebellar ataxic phenotype and isogenic male control mice of the same age were used. The animals were handled in accordance with current Spanish and European legislation governing the use of experimental animals (RD 53/2013 - BOE 08/02/2013 and 2010/63/EU), and all experimental procedures were approved by the Pablo de Olavide University ethics committee and the Junta de Andalucía (Animal Health Service auth. # 13/06/2017/080).

### Histological Procedures

#### Golgi-Cox Method and Dendritic Spine Analysis

Controls (*n* = 3) and *tbl* (*n* = 3) mutant mice were deeply anesthetized with an overdose of pentobarbital (80 mg/kg i.p.) and perfused intracardially with 4% paraformaldehyde (PFA) in phosphate buffer (PB 0.1M, pH 7.2–7.4). After dissection the brain was divided into two sagittal halves along the interhemispheric fissure and processed using a modified Golgi-Cox method (Bayram-Weston et al., 2016). Briefly, after 2 weeks in darkness in the Golgi-Cox solution (1.78% potassium dichromate, 1.78% mercuric chloride and 1.78% potassium chromate in distilled water) at room temperature (RT), the brain hemispheres were immersed for 24 h in 25% sucrose in Tris buffered saline (TBS, 0.1M pH 7.4). Sagittal frozen microtome sections (90 μm thick, Leitz) were obtained and collected in TBS. After a 5 min of treatment with 0.1% Triton X-100 in TBS, the sections were mounted on gelatin-coated slides and air-dried in the dark at RT. The sections were then rinsed in distilled water (1 min) and immersed in 25% ammonium hydroxide solution (Fluka, cat. 17093-1L), and after rinsing the sections in distilled water (1 min) they were transferred to Kodak Professional rapid fixer solution A for 20 min (Mychasiuk et al., 2013). After rinsing again with distilled water (1 min) the sections were dehydrated with ascending grades of ethanol (70%, 90% 1 min each, and 100% twice for 5 min each), cleared in xylene (2 min × 10 min) and mounted in DPX (Flores et al., 2005). Images were taken on a Zeiss Axioimager M1 microscope and the figures were prepared using Photoshop 8.0 software (Adobe^®^) with no additional correction.

Dendritic spines were analyzed as described by Pérez-Villegas et al. (2018). Briefly *Z*-stacks of completely filled, Golgi-Cox stained secondary dendrites of CA1 pyramidal neurons and dentate gyrus (DG) granule cells (optical section thickness = 0.5 μm) were visualized using a 100× oil-immersion objective with a numerical aperture of 1.74. The series of images were converted to RGB using the Fiji ImageJ software (W. Rasband, National Institutes of Health^1^) and then analyzed with the Reconstruct software^2^. Dendritic length, width and the length/width ratio were measured, and the spines were categorized according to steps 2 and 3 of the procedure in Risher et al. (2014). The data obtained were processed in Microsoft^®^ Excel (steps 4–6 of the same authors).

#### Immunohistochemical Procedures and Quantification

Controls (*n* = 3) and *tbl* (n = 3) mice were used. Mice were deeply anesthetized with an overdose of pentobarbital (80 mg/kg i.p.) and perfused intracardially with 4% PFA in PB. After dissection, the brains were fixed overnight at 4°C in the same fixative and they were then immersed in 30% sucrose in PB at 4°C until they sank. Frozen coronal microtome sections (30 μm thick) were collected in PBS, and immunostained using the procedure reported by Pérez-Villegas et al. (2018). The primary antibodies used were: a rabbit polyclonal antiserum against calbindin (CaBP, 1:10,000, Swant, CB-38); a rabbit monoclonal antibody against caspase-3 (1:400, Thermo Fisher, #700182); a rabbit polyclonal antibody against cleaved caspase-3 (Asp175) (1:500, Cell Signaling, #9661); a goat polyclonal antiserum against doublecortin (DCX, 1:250, Santa Cruz, sc-8066); a mouse monoclonal antibody against the HuC/HuD neuronal proteins (1:200; Thermo Fisher, A-21271); a rabbit polyclonal antiserum against the SV2A presynaptic vesicle protein (1:200, Synaptic Systems, #119002); a mouse monoclonal antibody against the vesicular glutamate transporter 1 (VGLUT1, 1:100, Millipore, mab5502); and a rabbit polyclonal antiserum against glutamate decarboxylase 65 and 67 (GAD65-67, 1:500, Millipore, AB1511). The secondary antibodies used were: Alexa Fluor^®^ 594 donkey-anti-goat (1:500, Invitrogen, A150132); Alexa Fluor^®^ 594 donkey-anti-mouse (1:500, Invitrogen, A21203); and Alexa Fluor^®^ 488 donkey-anti-rabbit (1:500, Invitrogen, A21206). The sections were counterstained with DAPI (1:5,000, Sigma, D9542) and images were acquired on an upright Zeiss Axioimager M1 microscope or on an upright Olympus FluoView 1000 confocal laser scanning microscope. The figures were prepared using the Photoshop 8.0 (Adobe^®^) software without additional corrections.

Immunoreactivity was quantified as indicated previously (Pérez-Villegas et al., 2018). Briefly, an alternating sequence of laser pulses was used to activate the different fluorescent probes during image acquisition. Images were acquired with a 60× oil-immersion objective at a numerical aperture of 1.42. Images from the hippocampal CA1 of control and *tbl* mice were obtained in the same session under similar conditions (laser intensities and photomultiplier voltages). Quantification of the fluorescent labeling density was performed offline with ImageJ and the size of the areas measured was determined automatically by defining outline masks based on the brightness thresholds from maximal projected confocal images. The control and *tbl* images of SV2A, VGLUT1, and GAD 65-67 expression in the CA1 area (38,725 μm^2^ of *z*-stacks made up of 9 slices each 0.5 μm thick) were captured as follows and expressed in arbitrary units: GAD 65–67: Green laser intensity 10%, with photomultiplier settings HV 760, Gain 1, Offset 8; VGLUT1: Argon laser intensity 10% with photomultiplier settings HV 773, Gain 1, Offset 5; SV2A: Green laser intensity 3.5%, with photomultiplier settings HV 680, Gain 1, Offset 30.

#### Transmission Electron Microscopy (TEM)

Control (*n* = 2) and *tbl* (n = 2) mice were deeply anesthetized with pentobarbital (80 mg/kg i.p.) and perfused intracardially with ice-cold 1% PFA, 1% glutaraldehyde and 0.02% CaCl_2_ fixative in PB. After dissection, the brains were stored overnight in the same fixative at 4°C and coronal slices (0.5–1 mm thick) of the brain were post-fixed in 2% OsO_4_ in PB, stained in block with 1% uranyl acetate in 70% ethanol, dehydrated and embedded in Durcupan (Fluka^®^). Ultrathin (50-70 nm) sections were obtained with a Leica UC6 ultramicrotome, collected in copper grids (150 and 300 mesh), and observed by TEM without counterstaining on a Zeiss Libra microscope at 80 kV (CITIUS, University of Seville).

**FIGURE 1:**
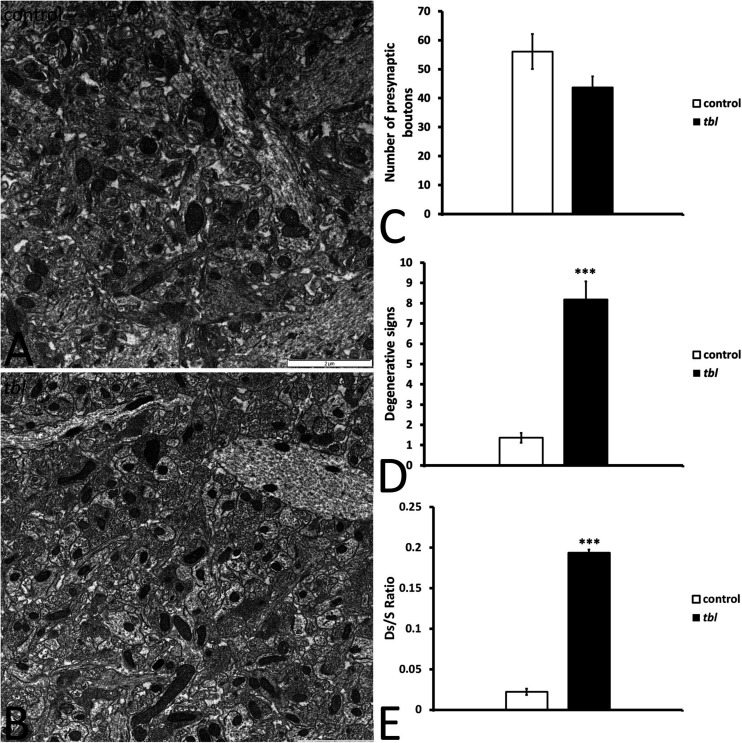
Panels **(A,B)** illustrate the transmission electron mosaic microphotographs used for the quantitative analysis. The only signs of degeneration within presynaptic terminals were counted, as described in the preceding figure (Figures 10C,D). No differences were found in the number of presynaptic terminals between the control and *tbl* CA1 (**C**, *p* = 0.1027245). However, there were significant differences in the signs of degeneration in the *tbl* CA1 presynaptic endings (**D**, ***p = 0.000014), as well as in the presynaptic endings with degenerative signs/synapses ratio (**E**, *** *p* = 0.0000008) relative to the control CA1.

**FIGURE 2:**
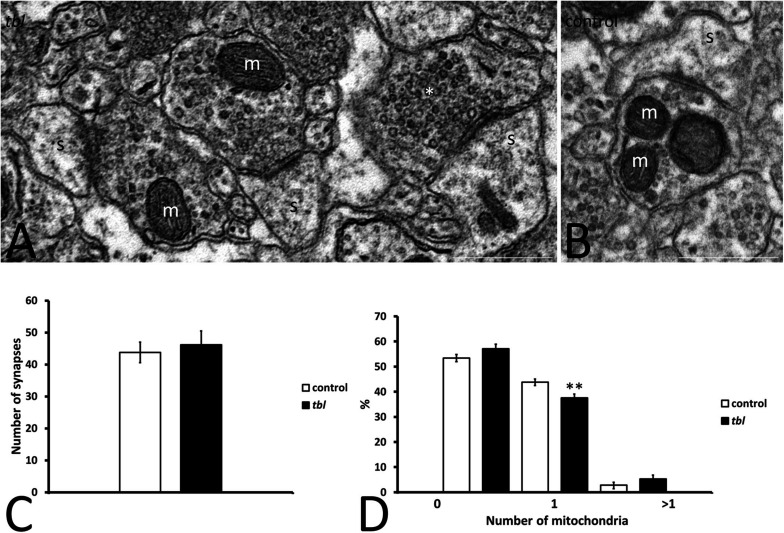
Microphotographs of part of the mosaics of the control **(B)** and *tbl* **(A)** CA1 used for quantitative analyses illustrating axospinous synapses (s) without mitochondria in the presynaptic ending (**A**, asterisk), or those containing one (**A**, m) or more **(B)** mitochondria. No differences were found in the number of presynaptic terminals in the control and *tbl* CA1 (**C**, *p* = 0.6546196). The only statistically difference found was in the number of presynaptic endings with one mitochondrion, which was lower in the *tbl* than in the control CA1 (**D**, **p = 0.0073196): s, postsynaptic dendritic spine. Bars = 0.5 μm **(A,B)**.

**FIGURE 3:**
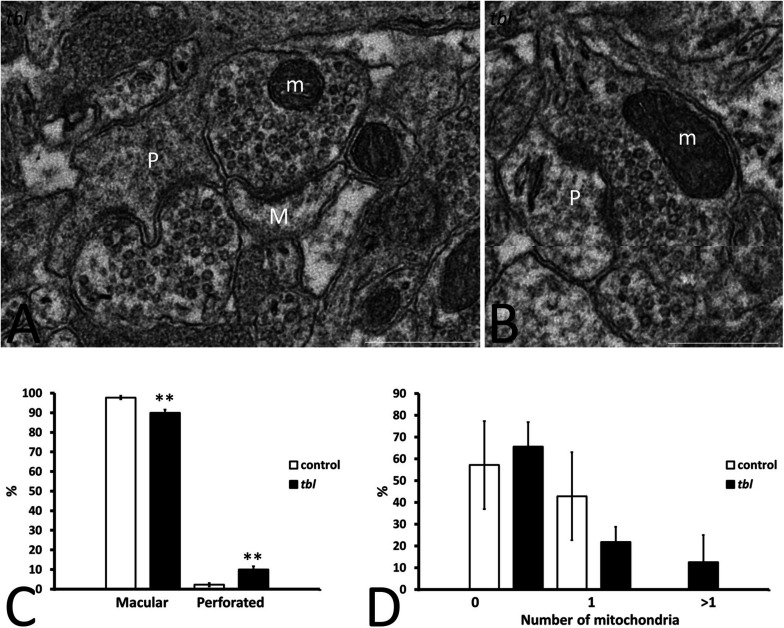
Microphotographs of part of the mosaics of the *tbl* CA1 used for the quantitative analyses of axospinous synapses **(A,B)**. A illustrates the criteria followed to assess the perforated (P) and non-perforated or macular (M) postsynaptic regions of axospinous synapses. Panel **(B)** shows a perforated synapse (P) whose presynaptic ending contains healthy mitochondria (m). Macular axospinous synapses were the most numerous in the control CA1 neuropil than in the *tbl* one (**C**, **p = 0.00202), while there were fewer perforated ones in the control CA1 neuropil than in *tbl* mice (**C**, **p = 0.00202). No significant differences were found in the number of perforated synapses with (**D**, 1 and >1) or without (**D**, 0) mitochondria within their presynaptic endings between the control and *tbl* CA1 neuropil (**D**, 0; *p* = 0.7218734; **D**, 1; *p* = 0.358118; **D**, >1; *p* = 0.350616). Bar = 0.5 μm **(A,B)**.

**FIGURE 4:**
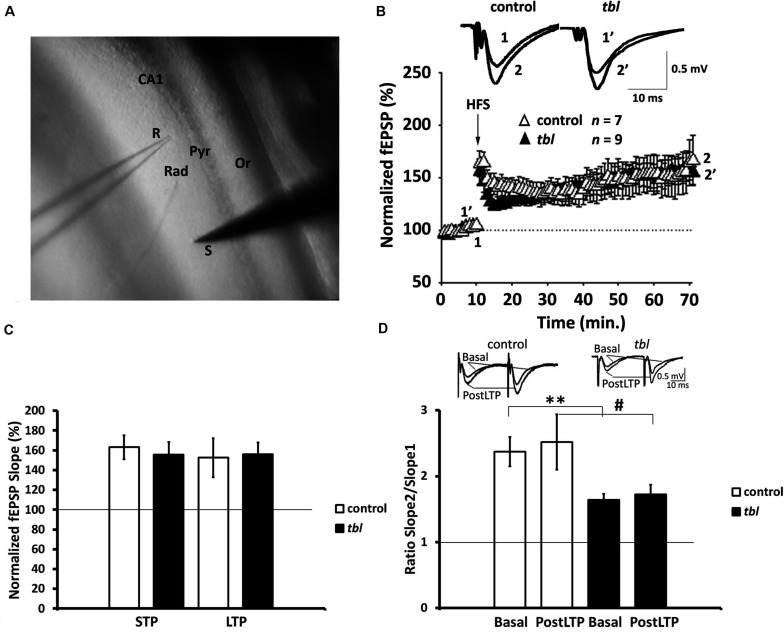
Long-term potentiation (LTP) is preserved in the Schaffer collateral-CA1 synapses of *tbl* mutant mice. **(A)** Stimulation and recording electrode configuration: S, stimulation; R, recording; Rad, *stratum radiatum*; Pyr, pyramidal cell layer; Or, *stratum oriens*. **(B)** Time course of the LTP protocol on the slope of the fEPSP in slices of control (white triangles) and mutant mice (black triangles). Traces show the fEPSP before (1, 1′) and 60 min after (2, 2′) the LTP protocol was applied. The symbols represent the average responses plotted every 60 s for each genotype. After 10 min of control recordings, a HFS train was applied as indicated by the vertical arrow. The number of slices for each genotype used (*n*) is indicated. **(C)** LTP and STP did not differ between the genotypes (*p* > 0.1, in both LTP and STP). **(D)** Paired-pulse ratio (PPR, slope 2/slope 1) traces show the PPF of fEPSP in control and *tbl* mice during basal stimulation and 55–60 min. after the HFS protocol was applied. A reduction in basal stimulation between the genotypes is evident (**p < 0.01), a difference that is not statistically significant 55–60 min after the HFS protocol (postLTP: ^#^*p* > 0.1).

Mosaic 3 × 3 (85 μm^2^ area) or 4 × 4 (170 μm^2^ area) microphotographs were obtained with the multiple image acquisition application of the Olympus iTEM software^®^. Images were obtained from ultrathin sections of the middle tier of the CA1 *stratum radiatum*, with 3 μm between each field to be sure that all counted axospinous synapses were different. The presynaptic terminals counted had clear synaptic vesicles, and an evident pre- and postsynaptic density in the plane of the section. The areas analyzed for counting measured: (i) 1,678 μm^2^ control and 1,767 μm^2^ *tbl* CA1 to assess the degenerative presynaptic profiles (Figures 1–3); (ii) 1343.18 μm^2^ control and 1235.55 μm^2^ *tbl* CA1 to quantify the number of mitochondria within the presynaptic endings (Figures 2–4); and (iii) 1343.18 μm^2^ control and 1007.1504 μm^2^ *tbl* CA1 areas to evaluate the macular and perforated axospinous synapses (Figures 1–3). All counts were done with the Fiji ImageJ software (W. Rasband, National Institutes of Health^3^).

### *Ex vivo* Recordings

#### Slice Preparation

Coronal slices (350 μm thick) containing the hippocampus were prepared and maintained according to standard procedures (Negrete-Díaz et al., 2007; Andrade-Talavera et al., 2012). Briefly, animals were anesthetized with isofluorane (2%), decapitated, and their brain was removed and placed in ice-cold extracellular Ringer’s solution (R, in mM): 124 NaCl, 2.69 KCl, 1.25 KH_2_PO_4_, 2 MgSO_4_, 1.8 CaCl_2_, 26 NaHCO_3_, and 10 glucose (pH 7.2, 300 mOsm). Coronal vibratome slices were then maintained in continuously oxygenated extracellular solution for at least 1 h before use. All experiments were carried out at RT (23–26°C).

#### Electrophysiology

Field excitatory postsynaptic potentials (fEPSPs) were recorded with low-resistance glass pipettes filled with the external solution and situated in the CA1 region of the hippocampus (Arroyo-García et al., 2018). Potentials were evoked by applying electric pulses through monopolar electrodes placed in the *stratum radiatum*. Synaptic field potentials were elicited at a frequency of 0.2 Hz, and the slope of the recorded fEPSP was calculated and used as a measure of synaptic strength. After a stable fEPSP baseline period of 10 min, a LTP protocol was applied by stimulating Schaffer collateral fibers with a train of pulses at a frequency of 100 Hz during one second (HFS) at the same strength as the test stimulus. Post-stimulation recordings continued for 60 min and LTP was successfully induced when the average fEPSP slope size (measured 50–60 min after HFS) increased by at least 20% relative to the baseline (100%). STP was measured as the maximum slope (peak) after HFS stimulation. A 40 ms paired-pulse stimulation protocol was used for pair pulse ratio (PPR) analysis. The fEPSPs were recorded at 2 kHz using an Axopatch 200B (Molecular Devices) apparatus and they were acquired at 10 kHz. All measurements were performed and analyzed in a strictly blind manner, with the genotypes of the animals revealed only after the electrophysiological experiments and their evaluation were complete.

#### Data Analysis

Data were analyzed using the Clampfit software (Molecular Devices) and unless otherwise indicated, they are presented as the mean ± SEM obtained using the Student’s-*t* test. The last 10 min of recording was used to estimate the changes in synaptic efficacy compared to the baseline. To measure the PPR, the slope of the 2nd fEPSP was divided by the slope of the 1st fEPSP.

### Behavioral Tests

#### Novel Object Recognition Test

Control (*n* = 5) and *tbl* (*n* = 5) mice were tested as described previously (Cubillos-Rojas et al., 2016). Mice were placed in a rectangular arena (55 cm × 40 cm × 40 cm) and two identical objects (A–A) were placed in the arena during the training phase (5 min). Short-term memory (STM) was assessed by comparing the amount of time spent exploring a novel object (B) relative to that spent exploring the familiar one (A). Twenty four hours after training, long-term memory (LTM) was tested by comparing the time spent by the mice exploring another novel object (A–C). The relative exploration of the novel objects was expressed as a discrimination index [DI = 5 (*t*_*novel*_ − *t*_*familiar*_)/(_*tnovel*_ + t_*familiar*_)].

#### T-Maze Test

Exploratory memory was tested in a T-maze over three consecutive days (10 trials per day and mouse, *n* = 10). The trials for spontaneous alternation measurement were performed according to the protocol described by Deacon and Rawlins (2006). Briefly, the mice were confined to the arm they choose first for 30 s. Thereafter, all the doors and the central partition of the T-maze were removed, and the animals were left to choose freely between the two arms. Control mice performed each trial in less than 2 min but owing to their ataxia, the *tbl* mice took between 3 and 5 min to finish each trial.

#### Morris Water Maze Test

Spatial learning and memory were also tested in five control and five *tbl* mice using a procedure similar to that proposed earlier by Morris (1984). A circular pool, 100 cm in diameter and 45 cm high, was filled to a depth of 13 cm with water (23 ± 2°C) made opaque by the addition of 0.01% TiO_2_. Four arbitrary N, S, E, and W points divided the pool into four quadrants and a 7 cm diameter platform was hidden 1.5 cm below the water surface in the N quadrant. Each mouse performed 4 trials per day, with an inter-trial interval of 30 min, and in each trial (one trial per quadrant) the mice were placed in a different quadrant with their nose facing the pool wall. All the experimental sessions were recorded with a digital camera and the time spent to reach the platform (escape latency) was the main variable assessed, considering the maximum trial time as 90 s. In the training session (1st day) mice were first placed in the NE quadrant and if that they did not successfully reach the platform, they were manually guided to it. In the acquisition period (2nd to 5th days), mice were considered that have correctly found the platform when they remained on it for at least 10 s (5 s for *tbl* mice). The retention interval was 7 days and thus, on the 12th day after the beginning of the experiments the mice were subject to a trial similar to previous ones but in which the platform was removed. The time spent by the mice in the pool area where the platform should be located was measured.

The *tbl* mice have a low performance on motor test (see Mashimo et al., 2009; Porras-García et al., 2013; Bachiller et al., 2015). Therefore, we have measured the swimming speed of control and *tbl* mice before the onset of the test. As it would be expected, the swimming speed was faster in control (mean = 0.07 ± 0.01 m/seg) than in *tbl* (mean = 0.026 ± 0.002 m/seg) mice. However, this lowest speed did not impede that *tbl* mice successfully reached the platform.

### Statistical Analysis

The statistical analyses of the data from behavioral tests and histological experiments were analyzed blind by EMP-V and MP-R. A two tailed Student’s *t*-test was used to compare the data from *tbl* and control mice. Any *p*-value less than 0.05 was considered significant, indicated as follows: ^∗^*p* < 0.05, ^∗∗^*p* < 0.01, and ^∗∗∗^*p* < 0.001. No significant values were indicated as follows: ^#^*p* > 0.05.

## Results

### Analysis of the Dendritic Spines on CA1 Pyramidal Neurons and DG Granule Cells

**FIGURE 5:**
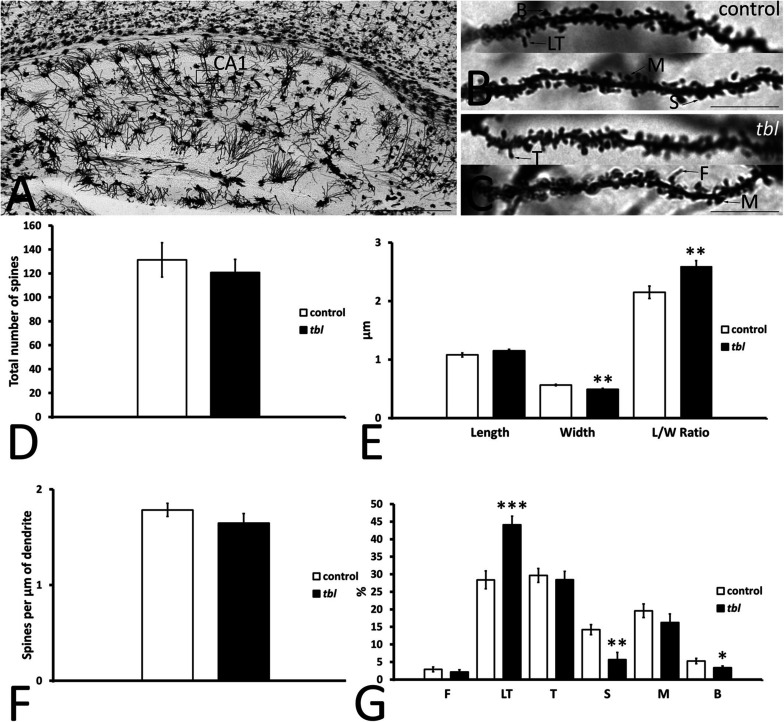
Microphotographs of parasagittal sections through a Golgi-Cox impregnated hippocampus **(A–C)**. **(B–C)** Illustrate the fully filled dendritic segments used to count and categorize the spines. **(D–G)** Graphical representations of the number of spines counted **(D)**, the differences in spine length **(E)**, spine width **(E)**, the spine length/width ratio (**E**, *L*/*W* Ratio) and in the density of spines **(F)** in the CA1 of control and *tbl* mice. The proportions of the dendritic spine types counted are also shown **(G)**. The smaller width of the spines and the higher length/wide ratio indicate the mainly immature spines in the *tbl* CA1. The most mature spines, mushroom spines (**G**, M) seem to be less abundant in the *tbl* CA1, although these values are not statistically significant, whereas a significant decrease in the number of mature stubby and branched spines is evident in the *tbl* mice (**G**, S and B). In addition, there is a significant increase in the number of immature thin long spines (**G**, LT) in the *tbl* CA1. The asterisks indicate significant differences in the Student’s *t*-test: **p = 0.002978 (**E**, width); **p = 0.005185 (**E**, *L*/*W* ratio); ***p = 0.0003845 (**G**, LT); **p = 0.002825 (**G**, S); *p = 0.0290842 (**G**, B). CA1, cornu ammonis 1; F, filopodia; LT, long thin spines; T, thin spines; M, mushroom spines; S, stubby spines; B, branched spines. Bars = 2mm **(A)**, and 10 μm **(B,C)**.

CA1 pyramidal neurons are easily distinguished by their triangular or pyramidal soma within the pyramidal cell layer, from which several basal and one or two primary dendrites arise. Primary dendrites and their branches spread through the *stratum radiatum* and the *stratum lacunosum-moleculare* where they receive distinct inputs (i.e., Schaffer collateral from CA3 pyramidal neurons and entorhinal afferents, respectively) (Amaral and Lavenex, 2007). Here we analyzed segments of the secondary dendrites of pyramidal neurons placed within the *stratum radiatum*, approximately at the same level at which the electrophysiological recordings were obtained (Figure 5A, open rectangle, see also Figure 4A). Dendrite segments 15–20 μm long that were completely filled by the Golgi-Cox metallic mercuric deposit were analyzed (Figures 5B,C), using previously described criteria to categorize and quantify the dendritic spines (Pérez-Villegas et al., 2018). Dendritic spines counts were made over a total dendrite length of 519.18 μm in control and 517.43 μm in *tbl* neurons. There was a slight yet not significant decrease in the number of spines counted (Figure 5D; control mean = 131.29 ± 14.35 vs. *tbl* mean = 120.88 ± 10.80; *p* = 0.29), and in the spines density on *tbl* pyramidal dendrites relative to the controls (Figure 5F; *p* = 0.14). However, there was a significant decrease in the spine width in the *tbl* pyramidal neurons relative to the control spines (Figure 5E; *p* < 0.01), which was responsible for the significant increase in the spine length/width ratio (Figure 5E; *p* < 0.01). Indeed, this shift was coincident with the significant decrease in the stubby and branched mature forms of *tbl* spines (Figure 5G; *p* < 0.01 and *p* < 0.05, respectively). Decreases that were accompanied by a significant increase in the immature long thin forms of spines on *tbl* pyramidal dendrites relative to controls (Figure 5G; *p* < 0.001).

**FIGURE 6:**
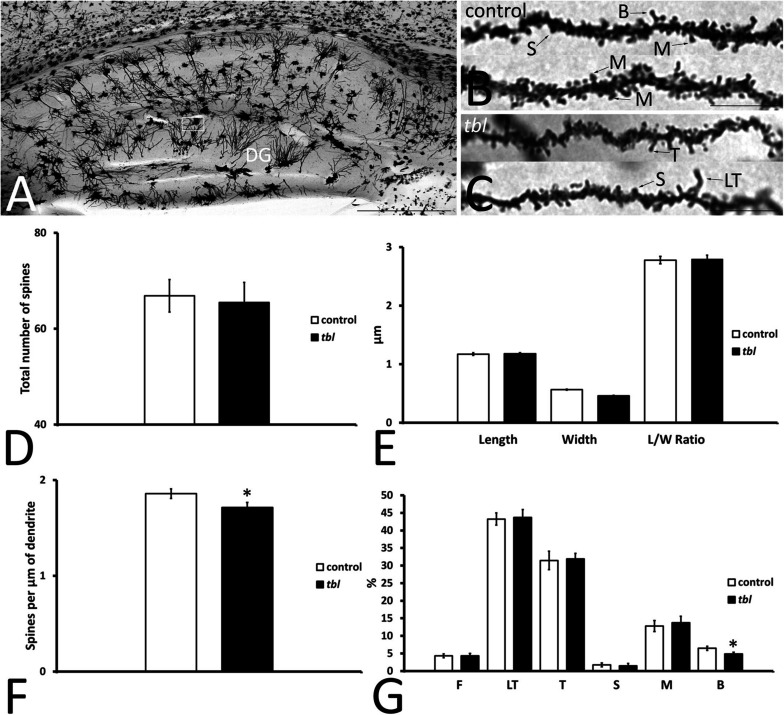
Microphotographs of parasagittal sections through the Golgi-Cox impregnated hippocampus **(A)**. **(B–C)** Show the completely impregnated dendritic segments of granule cells from the suprapyramidal blade of the dentate gyrus [DG: box in **(A)** represents the area used to count and categorize the spines]. **(D–G)** Graphical representation of differences in the number of spines **(D)**, the spine length **(E)**, in spine width **(E)**, the spine length/width ratio (**E**, *L*/*W* ratio), and the density of spines **(F)** in the DG of control and *tbl* mice. The proportions of the dendritic spine types counted are shown in **(G)**. No significant differences were found in the number (**D**; *p* = 0.7971753), length (**E**; *p* = 0.8019998) width (**E**; *p* = 0.7770199), and length/width ratio (**E**; *p* = 0.8729458) between *tbl* and control mice. However, the density of dendritic spines was lower in the *tbl* DG than in the controls **(F)**. The only significant differences in the type of dendritic spines was found in the branched spines, of which there were slightly fewer on *tbl* granule cell dendrites with respect to the controls **(G)**. The asterisks indicate significant differences in the Student’s *t*-test: *p = 0.0271162 **(F)**; *p = 0.0418517 (**G**, B). DG, dentate gyrus; F, filopodia; LT, long thin spines; T, thin spines; M, mushroom spines; S, stubby spines; B, branched spines. Bar = 2 mm **(A)**, and 10 μm **(B,C)**.

Granule cells are the projection neurons in the DG and their axons mainly end as mossy fibers to the CA3 field of the hippocampus (Amaral and Lavenex, 2007). Their dendrites arise from an ovoid cell body, spreading through the molecular layer and establishing dendritic trees in the suprapyramidal blade (dorsal leaf of Desmond and Levy, 1985) of the molecular layer, which are larger than those in the infrapyramidal blade (ventral leaf in Desmond and Levy, 1985). Here we analyzed the dendritic spines on granule cells in the outer third of the suprapyramidal blade (Figure 6A), which almost exclusively receives afferents from the perforant pathway (see figures 802 and 809 from Ramón y Cajal, 1904). Like the CA1 dendritic spines, 15–20 μm length segments of completely impregnated dendrites were considered (Figures 6B,C), analyzing total dendrite lengths of 544.99 μm for control and 503.12 μm for *tbl* mice. The number (Figure 6D; *p* = 0.80), the length and width, and the length/width ratio values (Figure 6E; *p* = 0.80, *p* = 0.78, and *p* = 0.87, respectively) were very similar between the control and the *tbl* mice, although the density of spines was lower on granule cell dendrites in the *tbl* DG than on control dendrites (Figure 6F; *p* < 0.05). Furthermore, while very long thin spines were observed on *tbl* granule cell dendrites (Figure 6C), the only significant difference among the different types of spines on granule cell dendrites was a decrease in the number of mature branched spines in the *tbl* mice relative to the controls (Figure 6G; *p* < 0.05).

### Immunohistochemistry

#### The Main Synaptic Vesicle Populations

**FIGURE 7:**
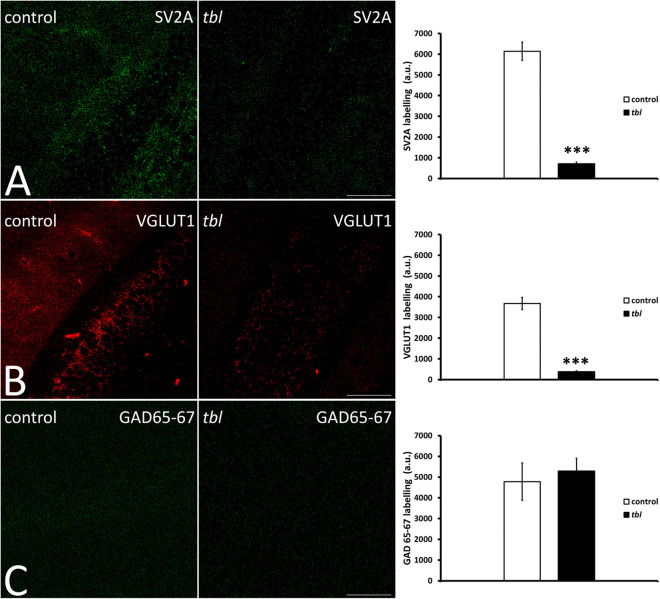
Laser confocal microphotographs **(A–C)** of coronal sections through the CA1 field of the hippocampus. **(A–C)** Z stacks of 9 slices (0.5 μm thick) illustrating the distribution of SV2A **(A)**, VGLUT1 **(B)**, and GAD 65–67 **(C)** immunoreactive synaptic vesicles in the control and *tbl* mice. Graphs showing the number of synaptic vesicles identified by SV2A immunoreactivity **(A)**, the synaptic glutamatergic vesicles identified by VGLUT1 expression **(B)**, and the GABAergic endings identified with the GAD 65–67 antibody **(C)**. The *tbl* CA1 possesses significantly fewer vesicles expressing SV2A (**A**, ***p = 4.0179^*E–*14^) and VGLUT1 (**B**, ***p = 1.68366^*E–*12^) as presynaptic markers, while no difference was found in GAD 65-67 expression (**C**, *p* = 0.3280439). Bar = 100 μm **(A–C)**.

Quantitative analysis of confocal laser microscopy images from the hippocampus demonstrated significantly weaker punctate immunolabelling of the integral membrane protein of presynaptic vesicles SV2A, and that of VGLUT1 considered to be a marker of glutamatergic synaptic vesicles (Figures 7A,B), in the *tbl* CA1 than in the control CA1, labeling fewer vesicles (Figures 7A,B; SV2A, *p* < 0.001; and VGLUT1, *p* < 0.001). By contrast, no significant differences in the intensity of presynaptic GABAergic GAD 65-67 immunoreactivity were found (Figure 7C; *p* = 0.39) between control and *tbl* CA1.

#### Immature vs. Mature Neurons in the Dentate Gyrus

**FIGURE 8:**
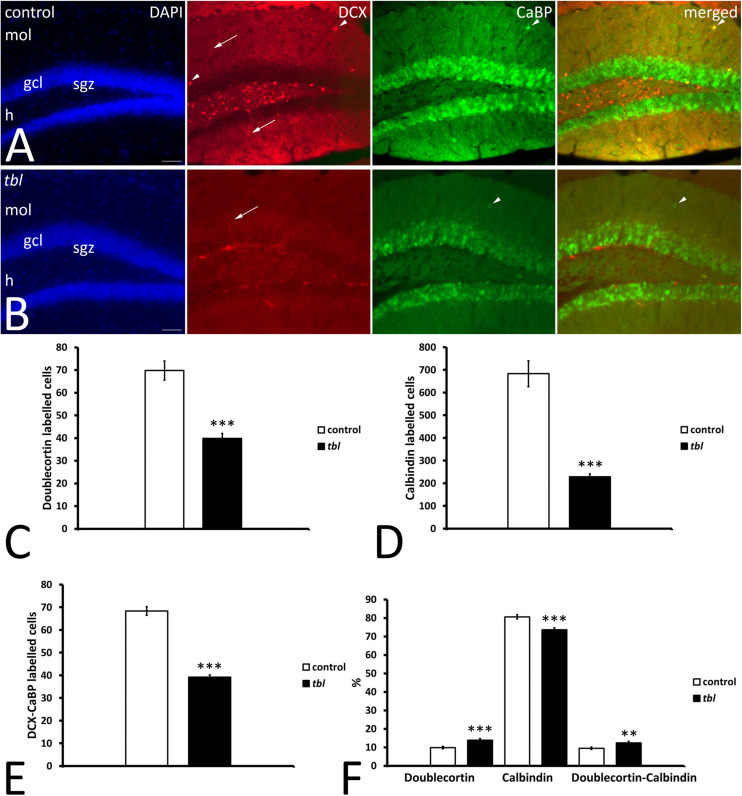
Microphotographs of coronal sections through the dentate gyrus of control **(A)** and *tbl* mice **(B)** illustrating DCX and CaBP immunoreactivity. DCX labeled cells occupy the subgranular zone, spreading their dendrites through the granule cell layer toward the molecular layer (arrows), while CaBP immunoreactive cells lie within the granule cell layer. A few labeled cells can be detected in the molecular layer (arrowheads). **(C–F)** Graphical representation of the number of DCX **(C)**, CaBP **(D)**, and DCX-CaBP **(E)** labeled cells, and the proportions of single and double-labeled cells **(F)**. The dentate gyrus of control mice possesses significantly more DCX, CaBP, and DCX-CaBP labeled cells than in *tbl* mice (**C**, ***p = 2.0063654^*E–*08^; **D**, ***p = 2.387923 ^*E–*10^; **E**, ***p = 1.665851^*E–*12^). However, the percentages of single and double labeled cells indicate that *tbl* mice possess significantly fewer calbindin immunoreactive neurons in the dentate gyrus than the controls (**F**, ***p = 0.0000242). By contrast, a higher proportion of DCX and DCX-CaBP labeled cells is found in the *tbl* dentate gyrus than in the controls (**F**, ***p = 0.0004682; and **p = 0.0060857, respectively), gcl, granule cells layer; h, hilus; mol, molecular layer; sgz, subgranular zone. Bar = 50 μm **(A,B)**.

Learning and memory are related to adult hippocampal neurogenesis (AHN) (Snyder, 2019). Therefore, to assess the possible effect of the HERC1 mutation on the maturation of postnatally born DG neurons, we assessed AHN using DCX and CaBP antibodies as markers of immature and mature neurons, respectively (Radic et al., 2017). DCX immunoreactive cells were mainly located in the subgranular zone of both the control and *tbl* DG (Figure 8). While some scattered DCX labeled cell bodies were also observed throughout the granule cells layer and the molecular layer of the control DG (Figure 8), they were virtually absent at these locations in the *tbl* DG. CaBP immunoreactive cell bodies were located within the granule cell layer, albeit with some exceptions (Figure 8). The qualitative observation of more CaBP immunoreactive cells in the DG of control mice relative to *tbl* ones was confirmed by the quantitative analysis. The counts of labeled cells made in 50 microphotographs from control and *tbl* mice, having each microphotograph an area of 356116.46 μm^2^ and containing the crest of the DG (“V” region), indicated that the number of DCX (Figure 8C; *p* < 0.001) and CaBP (Figure 8D; *p* < 0.001) labeled neurons was consistently higher in control than in the *tbl* DG. There was also a significant difference in the number of double-labeled cells between the control and *tbl* DG (Figure 8E; *p* < 0.001). However, when comparing the different cell types, there was a higher proportion of immature DCX-labeled neurons in the *tbl* DG relative to the control DG (Figure 8F; *p* < 0.001). Variation was also evident in the percentage of DCX and DCX/CaBP labeled cells, which was higher in the *tbl* DG than in the controls (Figure 8F; *p* < 0.001; and *p* < 0.01, respectively). Conversely, the percentage of CaBP expressing cells was significantly lower in the *tbl* DG (Figure 8F; *p* < 0.001). Hence, the *tbl* DG seems to possess fewer mature neurons than the control DG.

**FIGURE 9:**
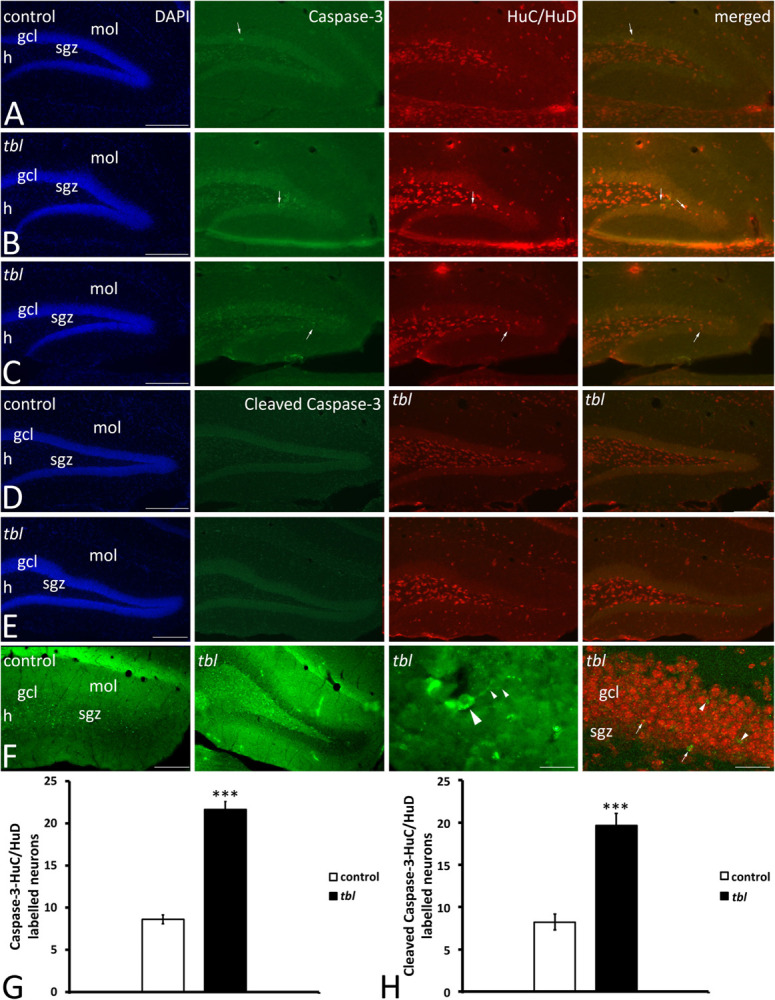
Microphotographs of coronal sections through the rostral **(A,B)** and middle **(C–F)** regions of the dentate gyrus of control **(A,C,E)** and *tbl* mice **(B,D,F)** to illustrate the immunoreactivity to caspase 3 and the neuronal marker proteins HuC/HuD. Almost all caspase-3 immunoreactive cells are located within the hilus, the subgranular zone and the granule cell layer, while HuC/HuD also labels interneurons located in the molecular layer. In the control DG it is possible to find caspase-3 labeled cells that do not co-express HuC/HuD (**A,C**, arrows), whereas in the *tbl* DG, caspase-3 immunoreactive cells also are labeled by the HuC/HuD antibody (**B,D**, arrows). Quantitative analysis demonstrates that the *tbl* DG has significantly more caspase-3-HuC/HuD (**G**, ***p = 1.902245^*E–*12^) and cleaved caspase-3-HuC/HuD double labeled neurons than the controls (**H**, ***p = 5.524^*E–*06^). gcl, granule cells layer; h, hilus; mol, molecular layer; sgz, subgranular zone. Bar = 200 μm **(A–F)**, and 30 and 60 μm **(F)**.

Our previous analysis on the immunohistochemical expression of the autophagy markers Beclin 1, LC3 and p62 in the CA3 field of the hippocampus showed that although the immunoreactivity of these proteins appeared to be stronger in the *tbl* than in control mice, almost all of these differences were not statistically significant (Ruiz et al., 2016). In neurodegenerative processes, the autophagy-apoptosis relationship is a mechanism potentially provoking neuronal cell death (Lee and Gao, 2009; Grishchuk et al., 2011; Napoletano et al., 2018). Furthermore, it was recently postulated that caspase-3 was less strongly activated by the pharmacologically attenuation of dysregulated autophagy in 6-OHDA treated cultured neurons (Chung et al., 2018). This autophagy-apoptosis link led us to study the distribution of caspase-3 and cleaved caspase-3 in the DG. Caspase-3 immunoreactive cells were randomly distributed throughout the DG, being most abundant in the polymorphic cell layer or hilus (Figure 9). Two qualitative differences stand out in caspase-3 immunoreactivity between control and *tbl* mice. First, the number and intensity of caspase-3 labeling was apparently greater in the *tbl* DG (Figures 9B,C) than in the control DG (Figure 9A). Secondly, while all caspase-3 positive cells were also immunoreactive to the neuronal marker HuC/HuD in the *tbl* DG (Figures 9B,C), some caspase-3 labeled cells did not express these neuronal proteins in control mice (Figure 9A). Cell counting ratified these qualitative differences and hence, there were significantly more cells expressing caspase-3 in the *tbl* than in the control DG (401.05 ± 46.82 and 172.56 ± 14.04, respectively; *p* < 0.001), as was the case for the number of neurons expressing caspase-3 in the DG (Figure 9G; *p* < 0.001). Similar results were found when observing the localization of cleaved caspase-3 in DG neurons (Figures 9D–F), and the number of double labeled neurons (Figure 9F) was significantly lower in the control than in the *tbl* DG (Figure 9H; *p* < 0.001).

In summary, present results strongly suggest that rather than alterations to the generation and differentiation of newly born cells in the DG, mature neuronal cell loss is greater in the *tbl* mutant mouse DG.

### Transmission Electron Microscopy (TEM)

**FIGURE 10:**
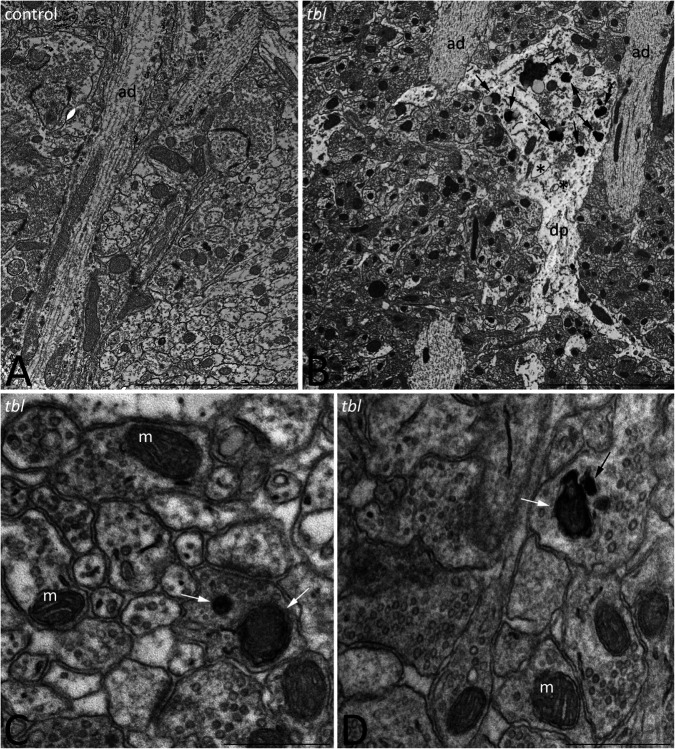
Transmission electron microphotographs through the *stratum radiatum* in the hippocampal CA1 region of control **(A)** and *tbl* **(B–D)** mice. Degenerative profiles (**B**, dp) whose cytoplasm is filled with altered vacuoles (**B**, asterisks), numerous lysosomes and necrotic debris (**B**, arrows), and mitolysosomes (**B**, arrowhead) are evident in the *tbl* mutant CA1. Mitophagic vacuoles and dark debris are often observed in axons and presynaptic terminals throughout the *tbl* CA1 neuropil (**C,D**, arrows). ad, apical dendrite; m, mitochondria. Bar = 5 μm **(A,B)**, and 0.5 μm **(C,D)**.

We previously described that pyramidal neurons of the CA3 field show signs of autophagy in the *tbl* hippocampus (Ruiz et al., 2016). Here, ultrastructural studies of the mid-regions of the *stratum radiatum* of the *tbl* CA1 (the same level at which electrophysiological recordings were made) identified signs of cell damage within the neuropil. Degenerative profiles containing large vacuoles with or without double membranes, dilated endoplasmic reticulum cisterns and lysosomes in different phases of maturity were evident, along with apical dendrites with a normal aspect (Figure 10B). Some of these lysosomes possessed a disorganized internal crest that resembled mitochondrial debris, mimicking mitolysosomes (Figure 10B). Mature mitophagosomes were also observed in axonal varicosities and presynaptic endings (Figures 10C,D), while no signs of evident damage were found in the cytoplasm of the postsynaptic CA1 dendritic spines of mutant mice. Quantification confirmed the significant differences for the signs of degeneration in presynaptic endings in the *tbl stratum radiatum* neuropil (Figure 1D; *p* < 0.001; and, Figure 1E; *p* < 0.001), while no significant differences between *tbl* and control mice were found in either the number of presynaptic endings (Figure 1C; *p* = 0.10) or in the size of neuropil area analyzed (control mean = 152.56 ± 10.38 vs. *tbl* mean = 160,63 ± 30.31; *p* = 0.81). Similarly, there were no differences in the ratio of the number of presynaptic endings relative to their area in μm^2^ (control mean = 0.37 ± 0.027 vs. *tbl* mean = 0.31 ± 0.029; *p* = 0.20), which would justify any errors in the differences in degeneration. Therefore, these observations reinforce and extend the earlier observations of damage (Ruiz et al., 2016) to the presynaptic endings in the CA1 of the *tbl* hippocampus.

The degree of presynaptic activity has been related to the presence of one or more mitochondria within the presynaptic terminal (Smith et al., 2016). Therefore, we counted the axospinous synapses with or without mitochondria at the presynaptic terminals in mosaic images of the control and *tbl* CA1 (control area mean = 149.24 ± 14.47 vs. *tbl* area mean = 137.28 ± 16.12; *p* = 0.59). Axospinous synapses were identified according the criteria of Peters et al. (1976), and the most abundant were those presynaptic endings possessing none (Figure 2A) or one healthy mitochondria (Figure 2A) per section, with synapses containing two or more mitochondria observed less frequently (Figure 2B). The number of axospinous synapses was similar in the control (394; mean = 43.78 ± 3.21) and *tbl* DG (416; mean = 46.22 ± 4.28; Figure 2C, *p* = 0.65). This quantitative analysis ratified that performed previously in the rat hippocampus by Shepherd and Harris (1998), with almost 50% of presynaptic endings in the control and *tbl* CA1 are devoid of mitochondria (Figure 2D). The difference in the number of presynaptic endings without mitochondria or with two or more mitochondria was slightly greater in the *tbl* than in control CA1 axospinous synapses, although these changes were not significant (Figure 2D; *p* = 0.12 and, *p* = 0.20, respectively). Notwithstanding, the decrease in the number of presynaptic endings possessing one mitochondrion in *tbl* CA1 axospinous synapses was significant when considered relative to their increase in the control CA1 (Figure 2D; *p* < 0.01).

Based on the structure of the postsynaptic density, two types of asymmetric axospinous synapses were observed in cortical and other brain areas: (i) non-perforated synapses (also named macular) with a single, continuous postsynaptic density (Figure 3A); and, (ii) perforated synapses in which the postsynaptic membrane density is divided by a small spinule (Calverley and Jones, 1987) (Figures 3A,B). Perforated synapses have been related to both plastic (Carlin and Siekevitz, 1983) and degenerative changes (Adams and Jones, 1982) in dendritic spines. In accordance with previous quantifications (Calverley and Jones, 1987), perforated synapses were always less numerous than macular synapsis in both control and *tbl* mice. No differences were found in the total number of axospinous synapses (control 175; mean = 25 ± 2.903 vs. *tbl* 268; mean = 33.5 ± 5.35; *p* = 0.20) or in the synapse densities in the CA1 neuropil (control mean = 0.196 ± 0.036 vs. *tbl* mean = 0.29 ± 0.063; *p* = 0.23). However, the number of non-perforated and perforated synapses between control and *tbl* DG varied, and there was a noticeable increase in perforated synapses in the *tbl* CA1 relative to the controls (control mean = 0.571 ± 0.02 vs. *tbl* mean = 3.375 ± 0.68; *p* < 0.01). In addition, the proportion of either type of synapse was significantly different between control and *tbl* DG. Thus, the increase in perforated synapses in *tbl* (Figure 3C; *p* < 0.01) was accompanied by an increase in the percentage of non-perforated synapses in the control CA1 (Figure 3C; *p* < 0.01). When we compare the presynaptic endings with or without mitochondria, no significant differences were found between the control and *tbl* mice CA1 (without mitochondria; *p* = 0.72; with one mitochondrion; *p* = 0.36; and with two or more mitochondria; *p* = 0.35; Figure 3D).

### CA1 Hippocampus Slice Recordings

The synaptic plasticity in both control and *tbl* mice were characterized by recording the fEPSPs evoked in CA1 hippocampal neurons (Figure 4A) by stimulation of Schaffer’s collaterals in the *stratum radiatum* of the brain slices (Figure 4A) (Andrade-Talavera et al., 2016; Arroyo-García et al., 2018). We used an HFS protocol to induce LTP, which induced clear STP and robust LTP in both control and *tbl* mice. The magnitude of potentiation (LTP) was expressed as the mean percentage of the fEPSP slope with respect to the baseline (set at 100%), and when quantified 50–60 min after stimulation it reached 152.6 ± 19.7% of the baseline in control mice *vs.* 155.4 ± 12.2% of the baseline in *tbl* mice (Figures 4B,C; *p* > 0.1). Likewise, STP was measured as the maximum slope found after HFS and it was 163.1 ± 12.2% vs. 155.8 ± 12.7% in control mice and *tbl* mice respectively (Figures 4B,C; *p* > 0.1).

In addition, the PPR was studied (the slope of the 2nd fEPSP divided by the slope of the 1st fEPSP slope) after LTP was induced by the HFS protocol (PostLTP), although it did not show differences in control (2.52 ± 0.42%) and *tbl* mice (1.73 ± 0.15%) (Figure 4D; *p* > 0.1), indicating there were no changes in the probability of release due the induction of LTP. However, while no differences in the magnitude of LTP and STP were found between control and *tbl* mice, and postsynaptic expression of this LTP was apparent in both groups of mice, paired-pulse facilitation PPF data indicated a clear difference in basal synaptic transmission between control and *tbl* mice. Thus, basal PPF was weaker in *tbl* than in control mice (*tbl*: 1.63 ± 0.09% vs. 2.37 ± 0.33% in control mice (Figure 4D; *p* < 0.01), indicating that a mechanism involved in PPF is at least partially impaired in *tbl* mice.

Hence, whereas synaptic transmission in the CA1 seems to be affected in *tbl* mice (PPF is reduced), this change in release does not affect the induction or expression of LTP in *tbl* mice, and both normal LTP and STP can be induced in these mice.

### Behavioral Studies

**FIGURE 11:**
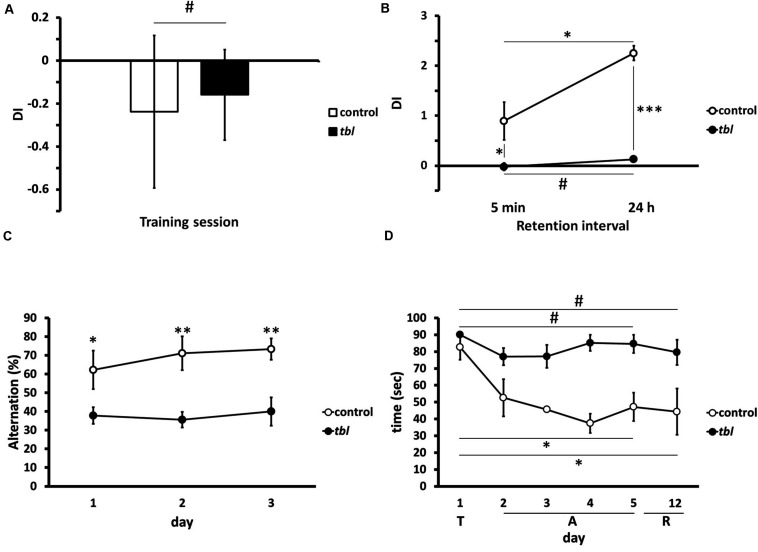
Graphical representations of the novel-object recognition **(A,B)**, the spontaneous alternation **(C)** and the Morris water maze **(D)** tests. Note the impaired recognition memory in *tbl* mice **(B)**, expressed as a Discrimination Index (**A,B**, DI), with respect to the controls at both short (**B**, 5 min, *p = 0.0337565) and long (**B**, 24 h, ***p = 0.000017) retention intervals after the training session (**A**, ^#^*p* = 0.428051). The differences in spontaneous alternation between control and *tbl* mice **(C)** clearly shows the loss of working memory in *tbl* mice (**C**, day 1 *p = 0.0406423; day 2 **p = 0.0058376; and, day 3 **p = 0.0047633). In the spatial reference memory task, and irrespective of the swimming speed of each group, control mice consistently decreased their escape latencies over the days (**D**, day 5 vs. day 1 *p = 0.0169509; and, day 6 vs. day 1 *p = 0.0459339), whereas *tbl* mice do not (**D**, day 5 vs. day 1 ^#^*p* = 0.2113249; and, day 6 vs. day 1 ^#^*p* = 0.1499300). T, training session; A, acquisition period; R, retention interval.

We used three tests to determine the effect of the *tbl* mutation on hippocampal memory tasks (for a review see Morris, 2007). In the novel-object recognition memory there was no difference between control and *tbl* mice during the training period (Figure 11A; *p* = 0.43), although there were significant differences in both STM (tested 5 min after training) and LTM (tested 24 h after training) with control mice displaying enhanced exploratory preference of the new objects relative to *tbl* mice (Figure 11B; *p* < 0.05 for STM; and, *p* < 0.001 for LTM). Moreover, there was also a significant difference between STM and LTM indicative of learning in control mice during the test (Figure 11B; *p* < 0.05).

Differences in the spontaneous alternation between control and *tbl* mice were significant from the first (Figure 11C; *p* < 0.05) to the last day of the experiment in which mice were tested (Figure 11C; *p* < 0.01 the 2nd day; and, *p* < 0.01 the 3rd day). Thus, while control mice increased their spontaneous alternation over time (62 ± 10.30% the 1st day; 71% 9.02 ± the 2nd day; and, 73 ± 5.67% the 3rd day), this was not the case for *tbl* mice that retained a spontaneous alternation below 50% (38 ± 4.44% the 1st day; 36 ± 4.16% the 2nd day; and, 40 ± 7.53% the 3rd day).

There were also differences in spatial learning and memory between *tbl* and control mice when analyzed in the Morris water maze test in. Thus, while control mice quickly learned to reach the hidden platform during the acquisition period (Figure 11D; *p* < 0.05), the escape latency to reach the platform did not shorten in *tbl* mice (Figure 11D; *p* = 0.211). Furthermore, there were clear differences in the escape latencies between control and *tbl* mice (*p* < 0.05) and the retention interval (*p* < 0.05); however, it could be argued that these differences were due to the faster swimming speed of control regarding *tbl* mice (see above in section “Morris Water Maze Test”), rather than the demonstration of damage of learning and spatial memory in *tbl* mutants. Notwithstanding the different swimming speed, the absence of a clear shortening in the latency of *tbl* mice along the test (Figure 11D; *p* = 0.211), clearly indicates that spatial learning and LTM were impaired by the *tbl* mutation.

## Discussion

The behavioral results presented here show that memory and learning processes involving the hippocampus (for a review see Morris, 2007) are impaired in *tbl* mice, which is perhaps not surprising given that several spontaneous mutations affecting cerebellar Purkinje cells also induce hippocampal damage (e.g., *leaner*, *reeler*, *staggerer*, and *weaver* mutant mice) (for a review see Grüsser-Cornehls and Bäurle, 2001; Porras-García et al., 2013). Moreover, ataxic mutant mice with primary cerebellar atrophy show altered spatial orientation and spontaneous alterations, while mice in which the cerebellum was not involved in ataxia did not show hippocampal-related learning difficulties (Lalonde, 2002). However, none of the cerebellar mutations with complete Purkinje cells loss produced evident hippocampal damage and the anomalous performance of these mice in hippocampal tasks was correlated to a putative alteration in the influence of the cerebellum on the cerebral cortex (Lalonde, 2002). As reported previously for the lateral amygdala (Pérez-Villegas et al., 2018), the alterations to dendritic spines observed here in the hippocampal CA1 and DG regions of *tbl* mice include signs of degeneration in the presynaptic terminal of CA1 axospinous synapses. By contrast, the weaker glutamatergic input to the CA1 in the presence of unaltered GABAergic input would justify the behavioral impairment, although unlike the lateral amygdala no significant alterations to STP and LTP were found in the *tbl* CA1.

From their seminal description by Ramón y Cajal (1888), dendritic spines were associated with neuronal communication. Through TEM, spines were related to excitatory synaptic transmission (Gray, 1959), and after the development of *in vitro* experimental methods, dendritic spines were consistently linked to learning and memory due to the plastic changes associated to LTP and/or LTD (for a review see Yuste and Bonhoeffer, 2001; Matsuzaki et al., 2004; Bailey et al., 2015; Grienberger et al., 2015; Segal, 2017). Three main morphological types of spines have been classified: mushroom, thin and stubby (Peters and Kaiserman-Abramof, 1969). Of these, the larger mushroom spines have been defined as stable spines and linked to memory, while stubby spines are the most abundant during postnatal development (Rochefort and Konnerth, 2012), and they have been classified as immature spines (Bourne and Harris, 2007). However, recent time-lapse super-resolution stimulated emission depletion studies demonstrated that stubby spines are mushroom spines whose neck is not so easy to visualize in conventional Golgi and EM analyses (Tønnesen et al., 2014), implying that stubby spines can be considered here as mature spines. Thin spines are silent spines, targets for the induction of LTP and hence, implicated in the ability to learn (Kasai et al., 2010). Immature long thin spines and filopodia are most abundant during synaptogenesis (Dunaevsky et al., 1999) and they finally evolve into mature spines (Segal, 2017). Variations in the number of spines and alterations in the spine morphology have been found in intellectual disability syndromes (IDs) (Levenga and Willemsen, 2012), in most of which a loss of spines is frequently coupled to a relative increase in immature spines, as in Patau’s and Down’s syndrome (Marin-Padilla, 1974, 1976; Purpura, 1975). However, while not all IDs are correlated with an evident decrease in spine number, they are related with a relative increase in immature spines, as occurs in autism spectrum disorders like the X-linked IDs (Levenga and Willemsen, 2012). Thus, studies on the hippocampus of the *Fmr1*-null mice, the most common murine model of Fragile X syndrome, did not provide clear information relative to the number, density, and maturity of the dendritic spines (Jawaid et al., 2018). Furthermore, studies of several mouse models of Rett syndrome also produced conflicting data on the number and density of hippocampal spines (Xu et al., 2014).

The dendritic spine counting performed here identified two different panoramas. There is an increase in the length of spines in the *tbl* CA1, which is correlated with an increase in immature long thin spines, and is coincident with a decrease in the stubby and branched mature spines, while the total number of spines and their density remains unchanged relative to the control CA1. Conversely, there is a significant decrease in spine density in the *tbl* relative to the control DG, principally due to a loss of mature branched spines. These changes are accompanied by differences in the proportions of the different morphologies in the postsynaptic regions of the spines. Four categories of chemical synapses have been reported in the nervous system based on their invaginations (Petralia et al., 2018). Of these, the simple axospinous synapse possesses a straight single postsynaptic density and it corresponds to those defined as non-perforated (Calverley and Jones, 1987) or macular (Marrone and Petit, 2002) synapses. Category 1 synapses correspond to the perforated synapses in which a small spinule devoid of a postsynaptic density protrudes into the presynaptic ending (Calverley and Jones, 1987). Perforated synapses have been defined as large stabilized mushroom spines that are involved in memory and learning as these spines are enhanced after LTP dependent learning (Marrone and Petit, 2002; Petralia et al., 2018). Furthermore, strengthening glutamatergic innervation induces the addition of AMPA receptors within the postsynaptic area (Park, 2018; Buonarati et al., 2019) that is responsible for the increase in postsynaptic density size (for review see Marrone and Petit, 2002; Yuste, 2010; Segal, 2017).

A clear decrease in perforated synapses in the CA1 region of *Fmr1* null mice coincided with their anomalous behavior (Jawaid et al., 2018), yet in the CA1 of *tbl* mice a significant increase in the number of perforated synapses was concomitant with a decrease of simple or macular synapses. The presence of more perforated synapses in the CA1 of *tbl* mice that perform poorly in learning and memory tests may be surprising, although the presence of perforated synapses has also been related to aging (Adams and Jones, 1982), reactive synaptogenesis after CA1 denervation (Marrone and Petit, 2002), and experimental dopamine depletion in the striatum (Anaya-Martínez et al., 2014). Furthermore, the permanence or increase due to *de novo* spinogenesis has been related to chronic denervation or long-term deprivation of the appropriate presynaptic inputs (Sotelo, 1978; Zuo et al., 2005). An increase in the number of immature spines was recently reported in the lateral amygdala of *tbl* mutant mice, even that of anomalous somatic spines (Pérez-Villegas et al., 2018). This increase in immature spines is associated with a dramatic decrease in glutamatergic inputs to this nucleus, yet no changes in NMDA and AMPA receptors were evident (Pérez-Villegas et al., 2018). Similar results were observed in the *tbl* CA1 region, where the decrease in glutamatergic synaptic vesicles contrasted with the stable NMDA (GluN1R) and AMPA (GluA1R) receptor subunit expression (data not shown). In addition, our TEM study adds new information regarding the presynaptic elements of axospinous synapses in the *tbl* CA1 neuropil, demonstrating significant signs of autophagy-mitophagy and a significant decrease in the number of macular synapses whose presynaptic terminals possess one mitochondrion. Mitochondria play a pivotal role in normal synaptic physiology, where they are responsible for glutamate synthesis (Waagepetersen et al., 2003), and mitochondrial ATP is essential for SV dynamics (Sudhof and Rizo, 2011). Indeed, synaptic terminals possessing mitochondria have fewer vesicles under conditions of LTP (reflecting an increase in neurotransmitter exocytosis), when postsynaptic spines show signs of sustained synaptic plasticity compared to those on presynaptic terminals in which mitochondria are absent (Smith et al., 2016). Thus, the decrease in the number of synapses containing mitochondria, together with the existence of mitophagy and evidence of degeneration in *tbl* presynaptic terminals, would at least partially explain the behavioral impairment of this mutant due to a loss of synaptic efficacy. Moreover, our studies on *tbl* hippocampal cultured neurons (Montes-Fernández et al., 2020) demonstrate a reduction in the ready releasable pool (RRP) and the resting pool (RP) of synaptic vesicles. A reduction in the RRP has also been described in the *tbl* neuromuscular junction, even before the onset of the ataxic syndrome (Bachiller et al., 2015), and in our cultures this coincided with a decrease in the total number of synaptic vesicles, a weaker clathrin expression and the absence of interactions between clathrin and mutated RLD1 (Montes-Fernández et al., 2020). Hence, the HERC1 mutation alters normal synaptic vesicles dynamics in presynaptic terminals.

The *tambaleante* mutation lies at the RLD1 of HERC1 (Mashimo et al., 2009). RLDs interact with ARF/Rab GTPases, and they have been implicated in intracellular membrane trafficking and clathrin dynamics (Sánchez-Tena et al., 2016). Therefore, the mutated HERC1 protein dysregulates clathrin coating and affects the endocytic pathway of synaptic vesicle recycling (Montes-Fernández et al., 2020), provoking a decrease in the RRP and RP, which explains the loss of glutamatergic synaptic vesicles in the *tbl* CA1. In addition, Rab endosomes are involved in sustaining mitochondria in axons, and the *Rab7a* mutation alters mitochondrial physiology and the anterograde axonal transport of mitochondria, contributing to Charcot Marie-Tooth disease (Cioni et al., 2019). Although a more in depth analysis of mitochondrial dynamics is needed in *tbl* neurons, the possibility exists that alterations to the interactions between Rab proteins and mutated HERC1 could alter late endosome-mitochondrial relationships; inducing mitochondrial dysfunction and eliciting the anomalous mitophagy that disrupts neuronal homeostasis (Martinez-Vicente, 2017). The E3 ligases in the UPS have been implicated in the maintenance of spine size, density and number, through regulation of AMPA receptor expression, or of NMDA receptor and RhoA activity (Hamilton and Zito, 2013; Mertz et al., 2015; Hamilton et al., 2017). Indeed, spinogenesis is also dampened when UPS activity is blocked (Hamilton and Zito, 2013). In our previous study on the lateral amygdala of *tbl* mice (Pérez-Villegas et al., 2018) we suggested that the increase in immature dendritic spines could reflect the increase in proteasome activity potentially caused by HERC1 overexpression (Mashimo et al., 2009). However, the absence of differences in the main glutamate receptors and the lack of evident signs of damage in the postsynaptic sites of axospinous synapses, together with the presence of presynaptic mitophagy, the smaller size of the RRP and RP of synaptic vesicles (Montes-Fernández et al., 2020, and present results), and the fewer presynaptic endings containing one mitochondrion, lead us to hypothesize that changes to spines in the *tbl* hippocampus could be a secondary effect. These changes may reflect the dysregulation of the normal synaptic transmission necessary for the formation and maintenance of dendritic spines (Yuste and Bonhoeffer, 2004; Turrigiano, 2008) rather than primary damage to the postsynaptic spine itself provoked by the mutation.

Autophagy plays a key role in neuronal homeostasis; it is essential for memory (Glatigny et al., 2019) and its dysfunction has been related to several neurological disorders (Ghavani et al., 2014; Lim and Yue, 2015; Nikoletopoulou et al., 2015). HERC1 overexpression elicits Purkinje cell death in association with extensive autophagy (Mashimo et al., 2009), as well as affecting other central nervous system neuronal populations (Ruiz et al., 2016). The data presented here extend previous data reported by Ruiz et al. (2016) on the hippocampal damage caused by the HERC1 mutation, demonstrating that the *tbl* DG possesses fewer postnatal mature CaBP hippocampal neurons, while new or immature DCX expressing cell populations seem not to be affected. Hence, the dysfunction in autophagy does not affect AHN. The regulation of apoptotic and necrotic cell death by autophagy is essential for homeostasis (Napoletano et al., 2018), the dysregulation of which has been implicated in several neurodegenerative diseases (Menzies et al., 2017; Alfaro et al., 2019). Indeed, recent evidence suggests that dysregulated apoptosis itself causes further cell death and apoptotic neuronal cell death (Chung et al., 2018). The increase in the caspase-3 and cleaved caspase-3 labeled neurons in the *tbl* DG is consistent with this proposal, opening the possibility that some cell death in the hippocampus may be driven by the *tbl* mutation, although less evident than in the cerebellum. AHN is implicated in learning and memory (Snyder, 2019), and an effect of the HERC1 mutation on such events cannot be ruled out. However, the scant evidence of largescale neuronal loss in the hippocampus compared to that seen in the *tbl* cerebellum cannot explain the behavioral impairments shown by *tbl* mice in this study. However, additional experiments using bromodeoxyuridine labeling and TUNEL staining to determine the exact rate of AHN and cell death will help us to define the role of HERC1 overproduction on adult neuronal renewal in the *tbl* DG.

It is possible that dysregulated macroautophagy-mitophagy occurs at the afferent axonal endings of the *tbl* CA1 and there is evidence that autophagy influences homeostasis of the presynaptic machinery (Vijayan and Verstreken, 2017; Lüningschrör and Sendtner, 2018). Thus, studies in the *Lurcher* mutant mouse demonstrated that Purkinje cell axons react earlier and more strongly to autophagy than the Purkinje cell bodies (Yue, 2007). Furthermore, in *Lurcher* mice lacking the *Atg7* gene, which encodes the autophagy-related protein 7, the axonal degeneration occurs before to and independently of Purkinje cell death (Yue et al., 2008). In fact, it is well established that the greatest number of neuronal autophagosomes accumulates in axon terminals (Maday and Holzbaur, 2014) and that axonal degeneration after injury is prevented by autophagy (He et al., 2016). Of the proteins involved in presynaptic autophagy, at least two would be affected by the *tbl* mutation. Firstly, the interaction of HERC1 with Rab GTPases (Sánchez-Tena et al., 2016; García-Cano et al., 2019), and among them Rab26 GTPase, is related to autophagy of the presynaptic machinery in the oldest or damaged synaptic vesicles (Binotti et al., 2015). Secondly, the mammalian target of rapamycin complex 1 (mTORC1) plays a key role in autophagy (Shimobayashi and Hall, 2014) and enhances autophagy and induces a loss of synaptic vesicles (Hernandez et al., 2012), when is inhibited. As mTORC1 activity is dampened by the *tbl* mutation (Mashimo et al., 2009; Bachiller et al., 2018), this decrease could explain the signs of autophagy and the reduction in the number of synaptic vesicles at the neuromuscular junction (Bachiller et al., 2015), in the lateral amygdala (Pérez-Villegas et al., 2018), in cultured hippocampal neurons (Montes-Fernández et al., 2020), and hippocampal CA1 presynaptic endings of *tbl* mice (present results).

Glutamatergic transmission plays a key role in LTP mechanism, and in the structural and molecular changes associated with learning (He et al., 2012). Postsynaptic glutamate receptors are thought to be essential for LTP induced spinogenesis (Yuste and Bonhoeffer, 2001; Lamprecht and LeDoux, 2004; Yuste, 2013; Rogerson et al., 2014) and LTP is frequently linked to the increase in the number of AMPARs in the postsynaptic membrane after NMDAR activation even in the CA1 region of the hippocampus (Kasai et al., 2010; Rogerson et al., 2014; Grienberger et al., 2015; Park, 2018; Buonarati et al., 2019). However, as was the case in the lateral amygdala (Pérez-Villegas et al., 2018), quantitative analysis of the *tbl* CA1 region did not demonstrate changes in the distribution of the GluN1 or GluA1 subunits and the differences in spine morphology cannot be related to alterations of the ionotropic glutamate receptors. As such, the similar number of ionotropic glutamate receptors contrasts with the reduction in the number of synaptic vesicles and in the VGLUT1 expression in the *tbl* CA1.

From our results, the altered learning in *tbl* mice seems to be parallel to a decrease in presynaptic glutamatergic input rather than to alterations in postsynaptic glutamatergic transmission. Indeed, we found electrophysiological differences in basal synaptic transmission in *tbl* mice when compared to control mice, with a lower PPF in *tbl* mice. Hence, the decrease in VGLUT1 expression and the lower number of vesicles seems to alter basal synaptic transmission at the level of the PPF in fEPSP recordings. Interestingly, these changes in presynaptic activity do not affect the STP (at 100 Hz) or LTP, indicating that STP or LTP do not account for the learning and memory deficits observed in *tbl* mice. As differences in PPF are observed (two pulses at 50 Hz), it is possible that while synapses in control and mutant mice share the same characteristics at high stimulation frequencies (above 50 Hz), these synapses behave differently at lower and less persistent stimulation frequencies, accounting for the learning and memory deficits observed in *tbl* mice. Although future *ex vivo* experiments will explain these differences, our study on cultured hippocampal neurons indicates that there are consistently more FM-143 de-stained synaptic vesicles in cultured neurons from control than *tbl* mice when were stimulated with 20 Hz trains after 40 and 700 action potentials. (Montes-Fernández et al., 2020).

In conclusion, the HERC1 mutation impairs hippocampal learning and memory in *tbl* mice. This learning deficit correlates with a decrease in the number of glutamatergic synaptic vesicles, signs of autophagy-mitophagy in presynaptic endings and alterations to basal synaptic efficacy despite STP and LTP remain unaffected. Therefore, the HERC1 E3 ligase protein, like other E3 ligase proteins, could contribute to the regulation of postsynaptic dendritic spinogenesis and to the homeostasis of presynaptic terminals. However, whether these alterations are due to the mutated RLD1 domain interfering with normal synaptic vesicles dynamics (Montes-Fernández et al., 2020) and/or altering the late-endosome-mitochondria relationship or to alterations in the proteostasis dysregulated by macroautophagy, or a combination of them remains unclear. Nevertheless, the data presented allow us to propose this *tbl* mutation as another model to study presynaptic homeostasis and its role in the maintenance of postsynaptic spines as a correlate for learning and memory processes.

## Data Availability Statement

The raw data supporting the conclusions of this article will be made available by the authors, without undue reservation, to any qualified researcher.

## Ethics Statement

The animal study was reviewed and approved by Pablo de Olavide University ethics committee and the Junta de Andalucía (Animal Health Service auth. # 13/06/2017/080). Written informed consent was obtained from the owners for the participation of their animals in this study.

## Author Contributions

JA: conceptualization, writing – original draft, and supervision. EP-V, MP-R, JN-D, GAT, and JA: methodology. EP-V, MP-R, JN-D, and JA: data acquisition and analysis. EP-V, MP-R, JN-D, RR, JR, GAT, AR-M, and JA: review and Editing. RR, JR, GdT, and AR-M: funding acquisition. All authors contributed to the article and approved the submitted version.

## Conflict of Interest

The authors declare that the research was conducted in the absence of any commercial or financial relationships that could be construed as a potential conflict of interest.

## Footnotes

1 https://imagej.nih.gov/ij/

2 https://synapses.clm.utexas.edu

3 https://imagej.nih.gov/ij/
